# A Slap at the Bacteriologists

**Published:** 1896-04

**Authors:** 


### A Slap at the Bacteriologists.

These are useful assistants, but they are tyrannical masters, and the results of a given treatment must, after all, be judged, not in the laboratory, but in the hospital ward and the sick room. A check must be imposed on garrulous bacteriologists who show a disposition to ride the cock-horse among us. We are grateful to them for such assistance as it may be in their power to render to medical science, but we cannot allow them to dictate to us what conclusions we are to draw from clinical investigation. Bacteriological statements are matters of inference, but clinical observations are facts; facts, too, which concern us more nearly than the interesting, but too often contradictory, deductions which foreign laboratory men foist upon us at the point of the scalpel.—*Medical Record.*

